# ASO Author Reflections: Preoperative Prediction of Postmastectomy Radiation Therapy After Neoadjuvant Systemic Therapy

**DOI:** 10.1245/s10434-019-07791-0

**Published:** 2019-09-04

**Authors:** Sanaz Samiei, Marjolein L. Smidt, Thiemo J. A. van Nijnatten

**Affiliations:** 1 Department of Surgery, Maastricht University Medical Center+, Maastricht, The Netherlands; 2 Department of Radiology and Nuclear Medicine, Maastricht University Medical Center+, Maastricht, The Netherlands; 3 GROW – School for Oncology and Developmental Biology, Maastricht University Medical Center+, Maastricht, The Netherlands

## Past

Immediate breast reconstruction and postmastectomy radiation therapy (PMRT) each play an integral part in multimodal breast cancer treatment. With the increasing use of neoadjuvant systemic therapy (NST) in early-stage breast cancer, the indications for PMRT are affected, and this contributes to the complexity of immediate breast reconstruction planning. The choice between immediate or delayed reconstruction is dependent on the preoperative need for PMRT. Residual axillary lymph node involvement after NST is the main determinant for PMRT since these patients have an increased risk of locoregional recurrence.^1^ However, axillary lymph node status in patients treated with NST is unknown prior to mastectomy, and the decision to perform immediate breast reconstruction is made without knowledge of the pathologic axillary outcome. In the case of immediate breast reconstruction, PMRT can not only adversely affect the cosmetic outcome of the reconstructed breast but can also increase the complication risks depending on the type of reconstruction.^2^,^3^ Therefore, preoperative identification of patients who do need PMRT after NST is advantageous to enable adequate shared decision making regarding the timing and type of reconstruction.

## Present

This study reports on the risk of positive sentinel lymph node (SLN) after NST in clinically node-negative breast cancer patients, and therefore the likelihood of PMRT.^4^ The ER−HER2+ subtype had the lowest rate (3.7%) of a positive SLN, whereas the highest rate (35.7%) was in the ER+HER2− subtype. In low-risk SLN positive patients (cT1-3N0 ER+HER2+, cT1-3N0 ER−HER2+, cT1-2N0 triple-negative), immediate breast reconstruction can be considered an acceptable option due to the decreased likelihood of PMRT. On the other hand, in high-risk SLN positive patients (cT1-3N0 ER+HER2−, cT3N0 triple-negative), the risks and benefits of immediate breast reconstruction should be discussed with the patient due to an increased likelihood of PMRT. For both situations, this study provides data for adequate shared decision making regarding the need for PMRT and timing of breast reconstruction (immediate or delayed).

## Future

Both breast reconstruction and PMRT are increasingly used, and the integration of these two important treatments requires a multidisciplinary approach and careful patient counseling. Our study provides data on low- and high-risk PMRT patients and can be included in the decision-making process. In high-risk SLN positive patients with a desire for immediate breast reconstruction, an SLN biopsy can be performed after NST prior to breast surgery in order to determine the pathologic axillary outcome and, consequently, the need for PMRT. If PMRT is required, the patient should be well-informed on the pros and cons of the combination of immediate breast reconstruction and PMRT, as well as the possibility of a delayed reconstruction. Finally, the decision to perform immediate or delayed reconstruction in the setting of PMRT must be individualized and must take into account the experience of the (plastic) surgeon and the expectations of the patient.
